# Performance of cowpea as influenced by native strain of rhizobia, lime and phosphorus in Samaru, Nigeria

**DOI:** 10.1007/s13199-017-0507-2

**Published:** 2017-09-14

**Authors:** S. K. Bello, A. A. Yusuf, M. Cargele

**Affiliations:** 1 Department of Soil Science, Faculty of Agriculture/Institute for Agricultural Research, Ahmadu Bello University, P.M.B. 1044, Zaria, Kaduna State Nigeria; 2 International Institute of Tropical Agriculture, P.O. Box 30772-00100, Nairobi, Kenya

**Keywords:** Biological nitrogen fixation, Integrated soil fertility management, *Bradyrhizobium* strain, Lime, Single superphosphate, Cowpea

## Abstract

The complimentary effects of a native rhizobia strain (SAMFIX 286), lime and single superphosphate (SSP) as components of ISFM were evaluated on the biomass, nodulation and N_2_ fixation of cowpea (*Vigna unguiculata* L.). Lime was applied at the rate of 250 kg (Ca(OH)_2_) ha^−1^, while SSP was applied at 30 kg P ha^−1^. The trial was carried out in a screen house with the treatments arranged in randomized complete block design. Results obtained show that the performance of SAMFIX 286 inoculated plants did not significantly (*p* < 0.05) differ from that of the un-inoculated treatment. Application of lime significantly increased root dry weight, shoot dry weight, nodule number and dry weight by 42.5%, 35.3%, 65.6% and 50%, respectively. Nodulation was significantly (*p* < 0.05) increased by SSP. The complimentary effect of lime with SSP significantly increased total shoot N concentration. Similarly, combined inoculation of SAMFIX 286 with lime and SSP increased N concentration by 31.9% and N derived from atmosphere (Ndfa) by 16.3% compared to the un-inoculated treatment. Inoculation of SAMFIX 286 with SSP was also effective on Ndfa by cowpea. It was concluded that lime and SSP were good combination with native rhizobia strain in improving cowpea nodulation and biological N_2_ fixation.

## Introduction

Low soil fertility is increasingly recognized as a fundamental biophysical cause for declining food security among small-farm households in sub-Saharan Africa (Sanchez et al. 1997). Poor soil fertility continues to represent huge obstacles to securing needed harvest (Sanginga and Woomer 2010). Reports also indicate that poor legume productivity on most small holder farms in sub-Saharan Africa is due to declining soil fertility and as a result of poor soil management practices (Chianu et al. 2011; Balume 2013). Thus, the integrated soil fertility management (ISFM) technique has been advocated as an approach to sustainably improve the soil fertility status of African soils. The set of soil fertility management practices included in ISFM are the use of fertilizer, organic inputs, and improved germplasm combined with the knowledge on how to adapt these practices to local conditions, aiming at maximizing agronomic use efficiency of the applied nutrients and improving crop productivity (Vanlauwe et al. 2010). While each component of ISFM can have a positive contribution to soil fertility and crop productivity, the aim of ISFM is to integrate multiple technologies in order to exploit complementarities among different protocols (Marenya and Barrett 2007).

Given the poor natural endowments of African soils, aggravated by poor management and sometimes non-appropriated soil practices, there is broad consensus that substantial increases in inorganic fertilizer use are necessary to restore and maintain the fertility of such soils to enhance their productivity (Minot and Benson 2009). Therefore, the use of inorganic fertilizers is part of the ISFM approach. The beneficial effects of combined organic and inorganic sources on soil fertility, crop yields, and maintenance of soil organic matter have repeatedly been shown in field trials (Chand et al. 2006; Nandwa 2003; Vanlauwe et al. 2002; Zerihun et al. 2013). Farmers use strategies such as application of organic and inorganic fertilizers, phosphate rocks, *Rhizobium* inoculants (Mungai and Karubiu 2011) and lime to address challenges of low soil fertility. The application of phosphorus (P) results in increased benefits in terms of yield and quality as it plays key roles in cellular energy transfer, respiration, photosynthesis and biological nitrogen fixation (Blackshaw and Brandt 2009; Mapfumo 2011; Jansa et al. 2011). Raising soil pH with lime results in greater activities of soil bacteria (Sanginga and Woomer 2010) which in turn increases soil organic matter decomposition, mineralization and nutrient cycling (Woomer et al. 1994) and also supplies Ca^2+^ for plant growth (White and Broadley 2003). However, organic and chemical fertilizers are not available to low-input African farmers majorly due to their associated high cost as generally those farmers are resource-poor. Thus, the introduction of selected beneficial microbes via biofertilizers could be considered as a practical, low cost strategy to improve crop yields in Africa.

Cowpea (*Vigna unguiculata* L. Walp) is one of the most important edible grain legumes in Africa contributing to food security and maintenance of environment for millions of small-scale farmers in sub-Saharan Africa (SSA) (Tarawali et al. 2002). Nigeria is the world’s largest producer of cowpea that supplies up to 40% of the daily protein intake of its constantly increasing population (Miko and Mohammed 2007). According to FAO data (2001–2010), Nigeria produces an average of 2.58 ± 0.31 million metric tonnes (AATF 2012). Thus, innovative research must be conducted to enhance the sustainability and improvement of cowpea production in Nigeria. Apart from the socio-economic value of cowpea, it has the ability to establish symbiosis with soil rhizobia to fix atmospheric N_2_ for growth, with the surplus N_2_ fixed benefiting succeeding crops (AATF 2012). The total of N_2_ fixed by grain legumes in SSA range from 11 to 201 kg N ha^−1^ for sole cropped cowpea (Giller et al. 1997), while in Nigeria the cowpea planted in the Northern Guinea savanna zone was estimated to fix 16–34 kg N ha^−1^ (Yusuf et al. 2007). Therefore, the biological nitrogen fixation (BNF) can reduce the need for N fertilizers, resulting in an economy estimated in US$ 3 billion per crop season (Mwangi 1994; Vinuesa et al. 2003; Shamseldin and Werner 2004; Nicolás et al. 2006; Shamseldin 2007). Inoculation with an effective and persistent rhizobial strain has numerous advantages, which include a marked reduction in the applied N fertilizer and the higher pod yield due to increased nodulation (Sanginga et al. 1994). Studies have shown that legumes dependent on symbiotic N fixation have high P requirement (IPNI 1999; Schulze et al. 2006; Raven 2012), which would be critical in acidic soil due to higher P fixation. Liming would enhance P availability to crops, and consequently improve the efficiency of legumes-rhizobia symbiosis in acidic soils. Based on this information, the main objective of this research was to test the effect of a native rhizobia strain, lime and phosphorus as ISFM components on cowpea growth in Samaru, Nigeria.

## Materials and methods

### Experimental site, soil sample collection and preparation

The trial was carried out in 2014 in a screen house at the Institute for Agricultural Research (IAR), Ahmadu Bello University (ABU), Samaru, Zaria located in the northern Guinea savanna (NGS) zone of Nigeria within longitudes 007^0^37.968′ E and latitudes 11^0^09.974′N, and an altitude of 698 m above sea level. Soil samples for laboratory analysis and screen house experiment were collected from the research farm (Field S13) of IAR. Field S13 is located on longitudes 007^0^36.865′ E and latitudes 11^0^11.018′N with an altitude of 701 m above sea level. On the basis of cropping history, it has been put into continuous cultivation with different mixtures of cowpea, groundnut and soybean over 20 years.

Soil samples were randomly collected using an auger at every 10 m^2^ within an area of 100 m^2^. The sampling depths were 0–5 cm for microbial analysis, 0–20 cm for analysis of soil physical and chemical properties and the screen house experiment. The soil samples were bulked, air dried and sieved through 2 mm mesh for routine soil analysis and 4 mm mesh for screen house experiments. Organic carbon (C) and total nitrogen (N) were determined from soils sieved through 0.5 mm mesh. For sowing in the screen house, experimental pots (12 cm in diameter) were filled with 10 kg of soil each.

### Application rates of the treatments and experimental layout

The rates of lime,, P and K used in this study were within the range (250 to 500 kg ha^−1^ for lime, 24 to 30 kg P ha^−1^, and 17 to 25 kg K ha^−1^) of current agronomic practices in the region. Briefly, lime (Ca (OH)_2_) was applied at 250 kg ha^−1^prior to sowing, single superphosphate (SSP) at 30 kg P ha^−1^, and muriate of potash (MOP) at 17 kg K ha^−1^ at sowing. Factorial combination was used to allot each treatment and treatment combinations to their respective pots (Table 1) except muriate of potash, which was applied to all the pots as a blanket treatment. The test crop was *Vigna unguiculata,* variety IT-89KD-288. Soil inoculation of the isolated native rhizobia strain, SAMFIX 286, was done by injecting 5 ml of the inoculant around the seedlings rhizosphere at 2 weeks after sowing. The experimental pots were arranged in randomized complete block design with three replicates.

**Table 1:** Treatments and treatment structure

Treatments	Cowpea
Inoculants	Un-inoculated
	SAMFIX 286
Lime	Lime
Single Superphosphate (SSP)	SSP
INTERACTIONS	
	Inoculant X Lime
	Inoculant X SSP
	Lime X SSP
	Inoculant X Lime X SSP

### Laboratory analysis

#### Soil chemical analysis

Soil chemical analysis was done using standard recommended methods. Soil pH was determined at soil:water ratio of 1:2.5 (IITA 1982), particle size distribution was determined using hydrometer method (Gee and Bauder 1986), total N was determined using the macro Kjeldahl digestion method as described by Bremner and Mulvaney (1982), organic carbon was determined using dichromate oxidation method (Nelson and Sommers 1982). The total P was determined using the wet oxidation method (ISRIC 1995) and the available P was extracted using Bray 1 (Bray and Kurtz 1945). The available P was finally estimated colorimetrically as described by Murphy and Riley (1962). Exchangeable bases were extracted using 1 N ammonium acetate and flame photometer reading was used to determine K, while atomic absorption spectrophotometer (AAS) was used to determine Ca and Mg. Exchangeable acidity was determined by titration method after extraction with 1 N KCl (Anderson and Ingram 1993). Effective cation exchange capacity (ECEC) was determined by summation of exchangeable bases and acids.

#### Treatment application and sowing

The application of lime was done prior to sowing. Seeds were surface sterilized prior to sowing by using 70% ethanol (C_2_H_5_OH) for 10 s, and 5% sodium hypochlorite (NaOCl) for 3 min and rinsed six times with sterile water. Distilled water was used to irrigate the pots throughout the experiment in order to reduce the nutrient load especially nitrates that may be supplied to the plants if tap water is used. The tap water could also be a source of high chlorine which is toxic to rhizobia. Five seeds per pot were sowed and later thinned to two seedlings per pot at 2 weeks after sowing.

#### Most probable number of native *Rhizobia* strains

The most probable number (MPN) was used to estimate the number of viable native rhizobia present in the experimental soil using the plant infection method. Hydroponic culture using Broughton and Dilworth N-free plant nutrient solution (Woomer et al. 1988) inoculated with serial dilution of the soil was used as a medium to grow cowpea for six weeks in growth pouches. The presence of nodules on cowpea at the end of the sixth week was used as an indication for the presence of rhizobia in the soil. The soil serial dilution was a five-fold six step dilution (5^−1^ to 5^−6^) (Woomer et al. 2010) as shown in in Eq. 1.

1 $$ Five- fold dilution=\frac{Volume of Sample\ \left(1\  ml\right)}{Volume of Sample\ \left(1\  ml\right)+ Volume of Diluent\ \left(4\  ml\right)} $$

Final rhizobia counts were determined using the MPN table with confidence intervals of *p* < 0.05 (Somasegaran and Hoben 1985).

#### Colony forming units of the native rhizobia strain

The MPN, expressed as colony forming units (CFU) per ml of inoculants was determined using the drop plate method as described by Woomer et al. (2010). Ten-fold, eight-step (10^−1^ – 10^−8^) serial dilutions of the native *Rhizobium* inoculant was made by adding 1 ml of yeast mannitol broth (YMB) culture to 9 ml of sterile water. 0.01 ml of the aliquots were transferred aseptically to dried yeast mannitol agar (YMA) plate having eight equal sectors being radially marked off on the outside bottom of the Petri dishes. The aliquots were allowed to dry by absorption into the agar. Thereafter, the plates were inverted and incubated for five days at 26–28° C to allow for the growth of rhizobium colonies. At the end of the procedures, the CFU count was calculated as shown in Eq. 2.

2 $$ MPN\ \left( CFU\ {ml}^{-1}\right)=\# Colonies\times Dilution Factor\times Inoculum Volume $$

where MPN, CFU, and ‘# colonies’ stand for most probable number, colony forming units, and number of colonies, respectively.

#### Plant sampling and tissue analysis

Plants (all plants per pot) were harvested at 8 weeks after sowing, early period of the reproductive growth stage, for tissue analysis. Total N was determined by using the macro Kjeldahl digestion method as described by Bremner and Mulvaney (1982).

#### Biological nitrogen fixation using the N difference method

Alongside the leguminous crop, maize was planted as a non-N_2_-fixing reference crop in order to estimate the amount of N fixed and percent N derived from BNF. Both the leguminous and maize crops were planted with same soil and under identical conditions in the screen house. After harvesting, the shoots were oven dried and analysed for total N to quantify N fixation. The amount of N fixed and percent N derived from BNF are estimated with the Eqs. 3 to 5 described by Mary et al. (1995).

3 $$ Total\ N\  in Plants\ \left( Total\ N\  Uptake\right)=\frac{Shoot\  Dry\  Matter Weight\times \%N\  in Plants}{100} $$

4 $$ {N}_2\  Fixed= Total\ N\  in Legume- Total\ N\  in Reference Crop $$

5 $$ \% Ndfa=\frac{Total\ N\  in Legume- Total\ N\  in Reference Crop}{Total\ N\  in Legume}\times 100 $$

Where, Ndfa means nitrogen derived from atmosphere.

#### Statistical analysis

Analysis of variance (ANOVA) was done using General Linear Model (GLM) procedure of SAS 9.3 Software (SAS 2011). Means were separated using Duncan’s Multiple Range Test at 5% significance level (*P* < 0.05).

## Results

### Characterization of soil and inputs

The results obtained from chemical analysis of the experimental soil (Table 2) showed that the soil was acidic; and had moderate P; high K; low organic carbon and N. The effective cation exchange capacity (ECEC) and exchangeable acidity (EA) were also low. Hence, the fertility level of the study-soil was low, however, response to P application alone is least expected since the soil’s available P is moderate. Since the soil pH was acidic, lime was applied to cushion the effect of acidity and enhance better performance of the native *Rhizobia* inoculant used in this study. The particle size distribution (PSD) showed that the textural class of the soil was Sandy Loam according to the United State Department of Agriculture Research Services (SPAW 2007). The most probable number (MPN) counts of rhizobia in the soil showed native cowpea rhizobia level of 7.5 X 10^3^ cfu g^−1^(Table 2). Analysis of the native rhizobia inoculant for number of viable cells showed that the strain had a CFU count of 3.3 X 10^8^. The quality standard which is 1.0 X 10^8^ ml^−1^ or g^−1^ of inoculant as recommended by the National Agency for Food and Drug Administration and Control (NAFDAC) in Nigeria was used in grading the inoculants as high or low in quality. Thus, SAMFIX 286 was graded as high quality inoculant.

**Table 2:** Characteristics of the soil used in the study

Property	Soil (Field S13)
pH_water_	5.4
Organic C (g kg^−1^)	1.4
Available P (mg kg^−1^)	10.0
Total P (g kg^−1^)	0.3
Total N (g kg^−1^)	0.5
Available nutrient (g kg^−1^)	
K	0.5
Ca	0.4
Mg	0.1
Exch. Acidity (cmol kg^−1^)	0.4
ECEC (cmol kg^−1^)	3.5
C:N	2.4
Particle size distribution	
Sand (%)	54
Silt (%)	28
Clay (%)	18
Textural Class	Sandy Loam
Cowpea’s rhizobia (cfug^−1^)	7.5 X 10^3^

### Cowpea biomass and nodulation

**Fig. 1:**
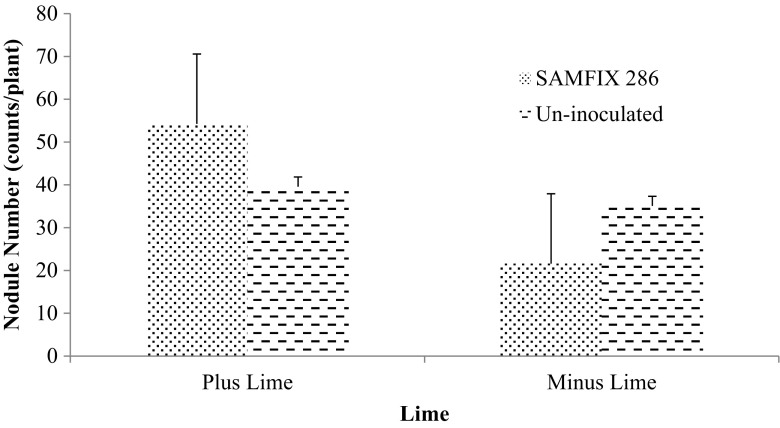
Interaction of Native *Rhizobia* Strain (SAMFIX 286) and Lime on Nodule Number (counts/plant) of Cowpea

The only significant interaction was found between inoculants and lime, and for only nodule number (Fig. 1). Inoculation with SAMFIX 286 significantly (*p* < 0.05) improved the nodule number in the presence of lime compared to SAMFIX 286 without lime and the un-inoculated control with and without lime. This could be related to the pH sensitivity of the native rhizobia strain (SAMFIX 286). The native rhizobia strain was collected in northern Nigeria but not on Field S13 where the present study soil was collected. However, the inoculation of SAMFIX 286 alone or with SSP and/or lime had no significant effect on biomass and nodulation whereas, application of lime had significant (*p* < 0.05) effect on both parameters (Table 3). Application of lime significantly increased root dry weight, shoot dry weight, nodule number and dry weight by 42.5%, 35.3%, 65.6% and 50%, respectively. The application of SSP significantly (*p* < 0.05) increased nodulation, i.e. nodule number and dry weight.

**Table 3:** Effects of Native Rhizobia Strain, Lime and SSP on Biomass and Nodulation of Cowpea

Treatments	Root Dry Weight (g/plant)	Shoot Dry Weight (g/plant)	Nodule Number (counts/plant)	Nodule Dry Weight (g/plant)
Inoculant				
Un-inoculated	1.16^a^	5.98^a^	37.33^a^	0.07^a^
SAMFIX 286	1.45^a^	6.28^a^	37.92^a^	0.08^a^
Lime				
Plus Lime	1.54^a^	7.05^a^	46.92^a^	0.09^a^
Minus Lime	1.08^b^	5.21^b^	28.33^b^	0.06^b^
Phosphorus				
Plus SSP	1.29^a^	6.81^a^	55.54^a^	0.12^a^
Minus SSP	1.33^a^	5.45^a^	19.71^b^	0.02^b^
SEM	0.13	0.61	5.09	0.01
Interactions				
Inoculant X Lime	NS	NS	*	NS
Inoculant X SSP	NS	NS	NS	NS
Lime X SSP	NS	NS	NS	NS
Inoculant X Lime X SSP	NS	NS	NS	NS

Means with the same letter within a treatment are not significantly different (*p* < 0.05) using DMRT (Duncan Multiple Range Test)

*Significant at p < 0.05﻿ an﻿d NS Not Significant at *p* < 0.05

### Cowpea tissue N concentration and biological nitrogen fixation

**Fig. 2:**
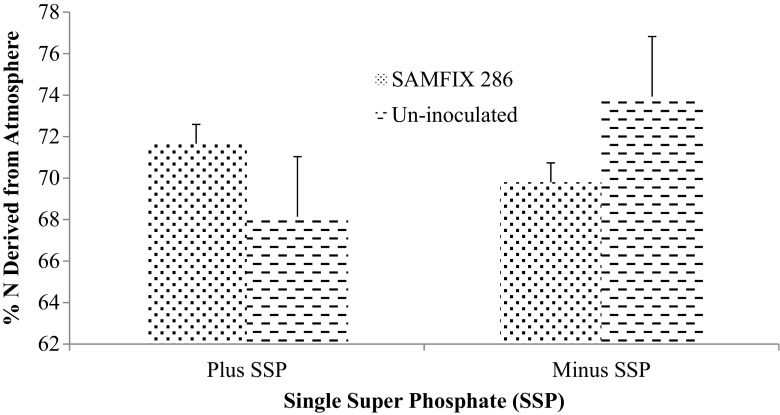
Interaction Effects of Native *Rhizobia* Strain and SSP on Percent (%) N Derived from Atmosphere

**Fig. 3:**
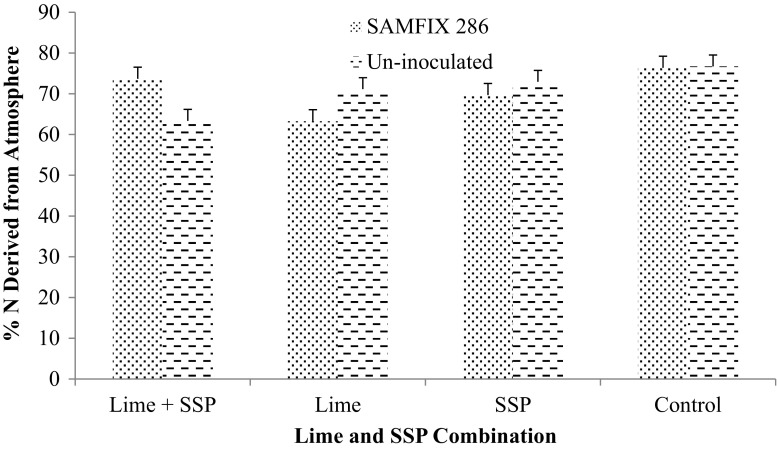
Interaction Effects of Native *Rhizobia* Strain, Lime and SSP on Percent (%) N Derived from Atmosphere

**Fig. 4:**
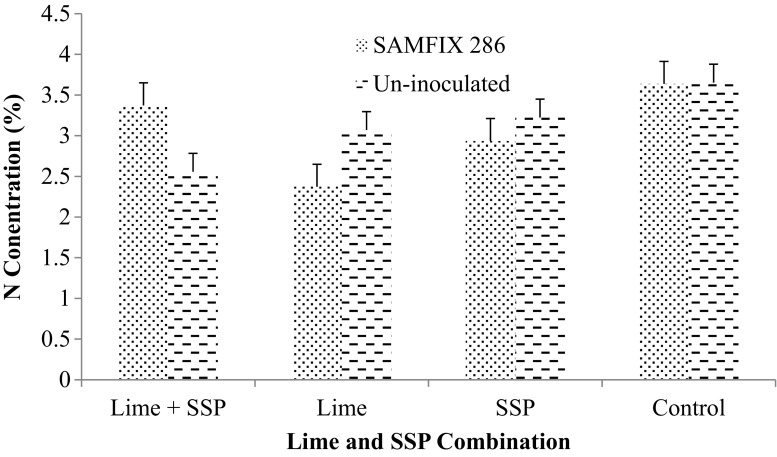
Interaction Effects of Native *Rhizobia* Strain, Lime and SSP on Shoot N Concentration of Cowpea

**Fig. 5:**
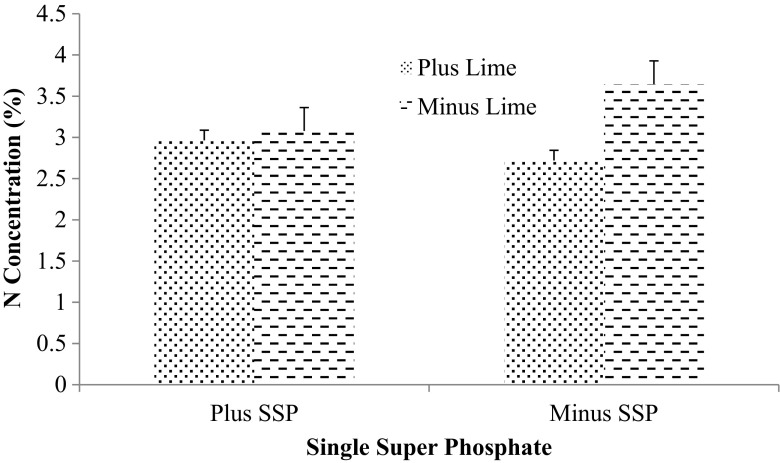
Interaction Effects of Single Super Phosphate and Lime on Shoot N Concentration of Cowpea

Significant interactions were observed between the inoculant and SSP as well as the combination of inoculant, lime and SSP for % Ndfa (Figs. 2 and 3). In addition, the interactions of inoculant, lime and SSP besides lime and SSP combination significantly increased N concentration (Figs. 4 and 5). SAMFIX 286 with SSP significantly improved % Ndfa, whereas in the absence of SSP, the un-inoculated treatment significantly (*p* < 0.05) improved % Ndfa compared to SAMFIX 286 without SSP (Fig. 2). The combination of SAMFIX 286 with lime and SSP significantly improved % Ndfa and N concentration compared to the un-inoculated treatment with lime and SSP (Figs. 3 and 4, respectively). The combined inoculation of SAMFIX 286 with lime and SSP increased N concentration by 31.9% and N derived from atmosphere (Ndfa) by 16.3% compared to the un-inoculated treatment. However, in the absence of lime and SSP, there were no significant differences between SAMFIX 286 and un-inoculated treatment. The interaction of inoculant and lime had no significant effect on N concentration, uptake and % Ndfa (Table 4). None of the interactions had significant effect on N uptake. SAMFIX-286 and SSP had no significant effect on N concentration, uptake and % Ndfa. Lime significantly decreased N concentration and % Ndfa.

**Table 4:** Effects of Native *Rhizobia* Strain, Lime and SSP on N Concentration, Uptake and Percentage N Derived from Atmosphere in Cowpea

Treatments	N Concentration (%)	N Uptake (mg/plant)	% Ndfa
Inoculant			
Un-inoculated	3.13^a^	183.68^a^	71.03^a^
SAMFIX 286	3.08^a^	183.06^a^	70.73^a^
Lime			
Plus Lime	2.84^b^	198.68^a^	67.85^b^
Minus Lime	3.36^a^	168.06^a^	73.92^a^
Phosphorus			
Plus SSP	3.02^a^	200.66^a^	71.87^a^
Minus SSP	3.18^a^	166.08^a^	69.90^a^
SEM	0.13	17.31	1.35
Interactions			
Inoculant x Lime	NS	NS	NS
Inoculant X SSP	NS	NS	*
Lime x SSP	*	NS	NS
Inoculant X Lime X SSP	*	NS	*

Means with the same letter(s) within a treatment are not significantly different (*p* < 0.05) using DMRT (Duncan Multiple Range Test).

*Significant at *p* < 0.05 and *NS* Not Significant at *p* < 0.05. % *Ndfa* N derived from atmosphere

## Discussion

The experimental soil had adequate natural population of indigenous rhizobia for cowpea nodulation. Amarger (2001) reported that rhizobia are widespread in tropical soil as a result of natural distribution and cultivation of legumes. Even more, cowpea is considered a promiscuous legume for its ability to nodulate with several rhizobia species and genera (Moreira 2008). Therefore, the establishment of foreign rhizobia strains in soils with substantial populations of indigenous rhizobia is considered difficult and response to inoculation unlikely (Abaidoo et al. 2007). Howieson and Ballard (2004) concluded that legumes will very often respond to inoculation where the rhizobial community is less than 100 cells g^−1^ of soil. Hence, the studied soil had high population of indigenous rhizobia for cowpea nodulation. This could explain the poor performance of the introduced strain when used alone without lime and SSP. In this case, the ineffectiveness of the inoculant was not attributed to a low quality of the product. Rather, the lack of response to inoculation could be ascribed to the presence of native effective rhizobial strain as previously shown (Ham et al. 1971; Vinuesa et al. 2003). In addition to the high population of indigenous rhizobia population, the study soil had low levels of organic matter, total N and it was acidic (Brockwell et al. 1991), which could have affected the symbiotic performance of the introduced rhizobia strain when used without lime and SSP.

The variable response to the co-application of inoculants and lime could be explained by the low application rate, which calls for the reassessment of lime recommendations in Nigeria. For example, in the soil acidity amelioration study of Peoples et al. (1995), lime was applied at the rate of 2500 kg ha^−1^ and this only raised the soil pH from 4.5 to 4.9. The increase in nodule number and dry weight of cowpea due to the application of lime was in concordance with the study of Bekere et al. (2013) where it was reported that lime application significantly increased number of nodules. Calcium supplied to plants through lime is essential component in symbiotic N_2_-fixation and nodule formation in legumes (Bambara and Ndakidemi 2010). The application of lime using Ca(OH)_2_ to bind excess H^+^ or Al^3+^ responsible for low soil pH while at the same time releasing Ca^2+^ as a nutrient could be said to have significant impact on cowpea growth in this study. The effectiveness of lime was further exhibited in nodule numbers when SAMFIX 286 was co-applied with it. The major influence of lime when applied in the soil is its ability to supply Ca^2+^ which is essential for plant growth (White and Broadley 2003) and neutralizing the toxicity effects of H^+^, Al^3+^ and Mn^2+^ in the soil (Staley and Brauer 2006). Consequently, lime also improved root and shoot dry weight. In the present study, there was no response to SSP application, in terms of root and shoot dry weight, due to the moderate level of P in the soil. This finding was in contrary to the study of Akande et al. (2010) who reported that application of SSP at the rate of 60 kg P/ha significantly increased the shoot dry weight of cowpea due to the low level of P in the study soil. However, P application synergistically worked with SAMFIX 286 to improve % Ndfa by cowpea. The quantity of P was sufficient to support nodule growth and activity; and the pH was above the critical limit of 5.0 for BNF (Howieson and Ballard 2004). Bhuiyan et al. (2008) reported that, the application of P along with rhizobia inoculant influences N_2_ fixation of legume crops. Phosphorus provides the mechanism for energy storage in the form of ATP and the transfer of that energy source to fuel vital plant functions such as N_2_ fixation (IPNI 1999). Nodulation, N_2_ fixation, and specific nodule activity are directly associated to P contribution (Abdulameer 2011).

The combined application of lime and SSP with SAMFIX 286 is an appropriate combination for improving N accumulation and N_2_ fixation by cowpea. The fundamental practice of liming acid soils is significant to the relationship between P and the symbiotic N fixation process (IPNI 1999). It improves the availability of soil P for plant absorption and creates a soil environment more favourable for beneficial bacteria such as the different strains of *Rhizobium*. Nodules develop when a root hair is infected by *Rhizobium* bacteria. Plant tissue develops around the infected area, forming the nodule and site of bacterial growth and the fixation of elemental N from the soil atmosphere (IPNI 1999).

## Conclusion

The effectiveness of lime and SSP varied in their performance on cowpea growth. Lime was effective on cowpea growth and could be recommended for cowpea cultivation on acidic soil to counter the negative effects of soil acidity; however, the appropriate rate requires further investigation. The application of SSP also positively influenced cowpea growth. The inoculant strain (SAMFIX 286) was less tolerant to low P conditions compared to other indigenous strains and thus, did not improve plant growth. Importantly, over the single effect of the treatments, the synergistic relationship between the treatments was found to be very important in improving the growth and yield of cowpea. It was concluded that lime and SSP were good combination with the native *Rhizobia* strain in improving cowpea nodulation and N_2_ fixation. Importantly, this study was limited to greenhouse evaluation and will require validation under field conditions at large scale before general recommendations could be made.
